# Purification of Prudu6 from Almond and Its Cross-Reactivity with Glym6 from Soybean

**DOI:** 10.3390/ijms26115425

**Published:** 2025-06-05

**Authors:** Changbao Hu, Qishu Luo, Lihua Zhou, Weichao Zhu, Kuan Gao, Qin Geng, Xin Li, Anshu Yang, Ping Tong, Zhihua Wu, Hongbing Chen

**Affiliations:** 1 State Key Laboratory of Food Science and Resources, Nanchang University, Nanchang 330047, China; 18189830072@163.com (C.H.); yihai104070030@163.com (Q.L.); 15679421009@163.com (L.Z.); 417900220189@email.ncu.edu.cn (W.Z.); 367900240001@email.ncu.edu.cn (K.G.); gengqin@email.ncu.edu.cn (Q.G.); zhizilixin@ncu.edu.cn (X.L.); yanganshu@ncu.edu.cn (A.Y.); tongping@ncu.edu.cn (P.T.); chenhongbing@ncu.edu.cn (H.C.); 2 College of Food Science and Technology, Nanchang University, Nanchang 330031, China; 3 Sino-German Joint Research Institute, Nanchang University, Nanchang 330047, China; 4 Jiangxi Province Food Engineering Research Center of Special Medical Purposes Intended for Allergic Population, Nanchang 330047, China

**Keywords:** Prudu6, purification, Glym6, cross-reactivity, epitope

## Abstract

Almond (*Prunus dulcis*) is a tree nut with high nutritional value that is widely cultivated and consumed globally. Prudu6, an 11S globulin, is one of the main allergens in almond, which can trigger a series of severe allergic reactions. To our knowledge, its correlation with Glym6, another 11S globulin, in terms of allergenicity has not yet been studied. In this study, natural Prudu6 was obtained by the optimized column chromatography method. Its structure was studied by the CD spectra, ultraviolet spectra and bioinformatics method. Then, WB and ELISA were performed to analyze the cross-reactivity. Prudu6 of high purity (>85%) was obtained by one-step chromatography. Strong cross-reactivity was found between Prudu6 and Glym6, which were also the main actors in the cross-reactivity between almond and soybean. For IgE in sera from almond-allergic patients, Glym6 demonstrated considerable affinity compared with Prudu6, while Prudu6 could hardly inhibit Glym6 in the soybean group. Three groups of epitope structures were found to be common in both proteins. These similar epitopes were regarded as the core structures causing the cross-reactivity between Prudu6 and Glym6.

## 1. Introduction

The almond (*Prunus dulcis*), a member of the Rosaceae family [1], is one of the most nutritionally significant tree nuts. Its health benefits are attributed to its rich composition of monounsaturated and polyunsaturated fatty acids, phytosterols, and polyphenols, including flavonoids. These bioactive compounds have been associated with reduced risk factors for cardiovascular diseases and diabetes [2,3,4], as well as antioxidant and anti-inflammatory effects [5,6]. Given these multifaceted health benefits, almond-derived products, such as almond oil, have gained considerable commercial and consumer interest [7,8,9].

As a member of the tree nuts that is one of the eight major allergens evaluated by the FAO and WHO, almond-induced allergies exhibit a broad global distribution. From the epidemiological perspective, the prevalence of almond allergy ranks fourth among the tree nut allergies [10]. Two related studies conducted in Mexico and Sweden reported severe allergies to almond among people of different age groups [11,12]. A considerable proportion of almond allergies occur in patients who are known to be allergic to other tree nuts [13]. The presence of such cross-reactive food proteins often poses challenges for the in vitro diagnosis and clinical management of tree nuts allergies [14,15], like almond allergy. Egger et al. [16] discovered the cross-reactivity between almond and tree nuts involving chestnut, hazelnut and walnut. In addition, foods other than tree nuts have also been confirmed to have cross-reactivity with almond, like peach, maze and peanut [17,18].

To date, seven groups of almond allergens have been included in the WHO-IUIS list of allergens and Prudu6 is the main allergen in almond. Comprised of a 40–42 kDa acidic α-chain and a 20 kDa basic β-chain [19,20,21,22,23], Prudu6 belongs to the cupin superfamily and is classified as an 11S seed storage globulin [24,25], which represent highly abundant proteins in both legumes and tree nuts, constituting more than half of the total protein content of almonds [26]. As an allergen with heat resistance, Prudu6 poses a serious threat to allergic patients and can lead to severe allergic reactions [27,28,29]. Therefore, the pursuit of high-purity Prudu6 is particularly necessary for research on allergy to almond. Meanwhile, Prudu6 may play an important role in the cross-reactivity between almond and other foods.

Simply speaking, the cross-reactivity among foods occurs because the IgE in the serum of allergic patients can recognize the different allergen epitopes in different foods. That is, the material basis of cross-reactivity is the shared similar epitope structure among allergens. Allergens belonging to the same protein family often have similar three-dimensional structures due to their characteristics, which also indicates great cross-reactivity potential among different allergens within the same protein family.

As one of the major contributors to the protein content of soybean (*Glycine max*), Glym6 is regarded as a potential diagnostic marker for severe allergic reactions to soybean [30,31]. Although preliminary investigations into the cross-reactivity were performed among almond as mentioned above, related information about soybean (*Glycine max*) was not explored. Moreover, the role played by Prudu6 and Glym6 (the main allergen in soybean [32,33]), which both belong to the 11S globulins, in this cross-reactivity remained unknown. In this research, we optimized a purification method for the major almond allergen Prudu6. Afterwards, the cross-reactivity between Prudu6 and Glym6 was systematically evaluated through serological methods. Combined with the epitopes, the multi-dimensional structures of Prudu6 and Glym6 were characterized to reveal the core molecular mechanism.

## 2. Results

### 2.1. Purification and Identification of Prudu6

**Figure 1:**
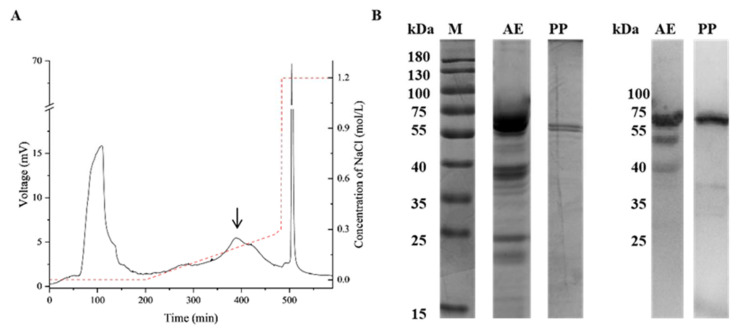
Purification of Prudu6. (**A**) Fragments of whole almond protein extract separated by anion-exchange chromatography; (**B**) SDS-PAGE (left) and Western blot (right) analysis of almond extracts using the sera of an almond-allergic patient. Lane M, protein marker; lane AE, almond extract; lane PP, purified protein.

After one-step chromatography, Prudu6 appears in the area indicated by the arrow (Figure 1A), the band distribution and IgE reactivity of the corresponding purified protein were demonstrated by SDS-PAGE and WB, which both showed one band with a molecular weight of around 60 kDa (Figure 1B). The results were consistent with the almond extracts in the corresponding band. Due to the similar molecular weight and the ability to bind to the IgE in the sera of patients allergic to almond, the purified protein was initially regarded as Prudu6.

To further prove the specific identity of the purified protein, the strip at 60 kDa was cut for mass spectrometry. The top ten matched peptides are showed in Table S1. The first accession E3SH28, which is the UniProt ID of Prudu6, had a significantly higher score than the others, with 30 specific peptides and 75% coverage. All this information indicated that the purified protein was Prudu6 (Table S3).

Through the optimization methods determined in our research, 4 mg of natural Prudu6 can be obtained from 2 g of almond defatted powder, with 85% purity.

### 2.2. Characterization of Prudu6 and Glym6

By comparing the sequence and spatial structure, 43.51% similarity was observed in the sequence, with a root mean square distance (RMSD) value of 0.509 in the spatial structure (Figure S1). Similar regions in the spatial structure were primarily concentrated in the center composed of a β-barrel structure (Figure S1), which was a characteristic feature of the cupin superfamily and represented a highly conserved structure in this class of proteins [34].

Generally speaking, in the context of predicting the cross-reactivity among allergens, it is known that the similarity of peptide sequences plays an important role: the higher the similarity, the stronger it is [35]. Cross-reactive allergens exist with a primary amino acid sequence similarity of more than 70% [36], which did not conform to our results. This might be attributed to the fact that most of the current cross-reactions research focused on proteins such as 2S albumins and lipid transfer proteins (LTPs), which are all proteins with molecular weights lower than 15kDa and simple spatial structures [37,38]. As 11S globulins, the molecular weights of Prudu6 and Glym6 can reach four or more times that of 2S albumins, with more complex spatial structures. Therefore, the conclusion regarding the sequence similarity is not applicable in the cross-reaction study of globulins.

The most widely used estimator of structural similarity is the RMSD between equivalent atoms, calculated using the optimal superposition of the two structures being compared. On average, smaller RMSD values are correlated with protein structure pairs determined at higher resolution [39].

**Figure 2:**
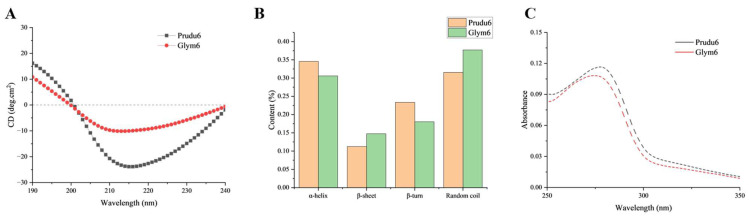
Secondary and tertiary structure analysis of Prudu6 and Glym6. (**A**) CD spectra. (**B**) Percentage of secondary structure units. (**C**) UV absorption.

The conformational structures of Prudu6 and Glym6 were characterized using the CD and UV spectra. The CD spectra were determined to analyze the secondary structure of the allergens (Figure 2A), with the percentage of each secondary structure unit (Figure 2B). It can be seen that at about 217 nm, the characteristic peak of the β-sheet, Prudu6 and Glym6 had high peak intensity. This is consistent with their characteristic cupin domain as 11S globulins, which is filled with a β-sheet [40]. The similar change curve in the UV absorption (Figure 2C) suggested similar aromatic residue compositions and comparable tertiary environments (e.g., folding or surface exposure of aromatic side chains).

Combined with the sequence similarity and RMSD value, the results of the CD and UV spectra indicated that Prudu6 and Glym6 had high similarity in their overall structure.

### 2.3. Cross-Reactivity Between Prudu6 and Glym6

**Figure 3:**
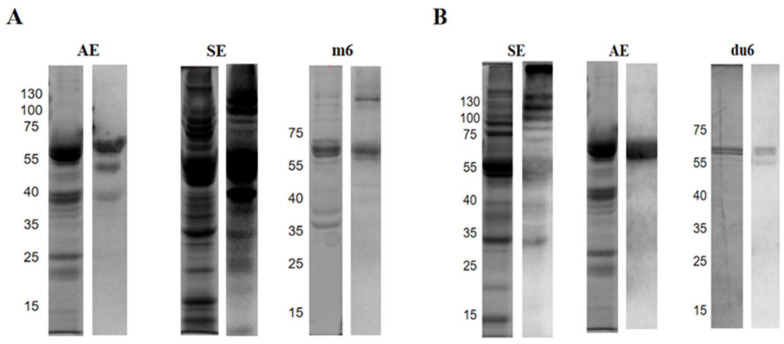
Cross-identification of Prudu6 and Glym6. SDS-PAGE (left) and WB (right) in sera from almond-allergic patients (**A**) and soybean-allergic patients (**B**). AE: almond extract; SE: soybean extract. du6, m6 represent Prudu6, Glym6.

Sera from patients with almond allergy or soybean allergy, respectively, was used to evaluate the cross-reactivity. The allergen recognition pattern of different sera was demonstrated by WB. As for the almond and soybean extracts, obvious recognition occurred in both groups of sera; especially, the soybean extracts had a strong IgE binding reaction to the sera of almond with a high molecular mass range (Figure 3A) where globulins mainly existed, including Glym5 and Glym6 [41,42]. Only a single band (around 60 kDa) in the almond extract was recognized by IgE in the soybean sera (Figure 3B), which suggested the sensitization of Prudu6 in patients with soybean allergy. The same operation was applied to Prudu6 and Glym6, and an obvious recognition response was shown, as we expected, which indicated the significance of Prudu6 and Glym6 in the cross-reactivity between almond and soybean.

**Figure 4:**
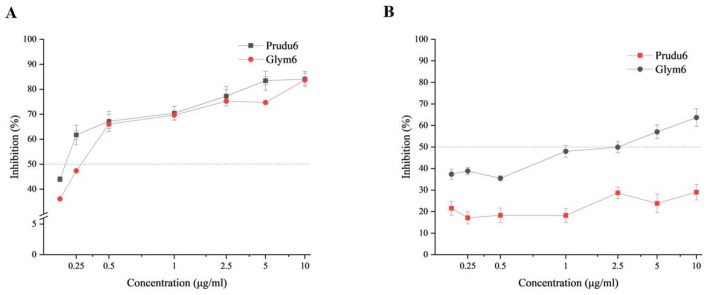
Affinity comparison of Prudu6 and Glym6 by indirect inhibition ELISA. (**A**) Group of sera from almond-allergic patients with Prudu6 as the coated protein. (**B**) Group of sera from soybean-allergic patients with Glym6 as the coated protein.

An ELISA assay was performed to further estimate the degree of affinity of Prudu6 and Glym6. In the case of almond-sensitized patients, the binding of IgE to Prudu6 was effectively inhibited by Prudu6 at all the used concentrations, and Glym6 demonstrated an inhibition rate curve that was strikingly similar to that of Prudu6 (Figure 4A). This high level of similarity in terms of inhibition indicated that Prudu6 and Glym6 had considerable allergenicity for patients allergic to almond and that the two allergens share completely common or similar epitopes. As to the soybean group, at most concentrations, Prudu6 was a substantially less potent inhibitor compared with Glym6, while at the highest concentration, its ability to inhibit was still weak (Figure 4B). These results showed that Glym6 was a more competitive and efficient inhibitor than Prudu6.

From the WB and ELISA analysis, Prudu6 and Gly m6 were demonstrated to be the main allergens causing the cross-reactivity through the above studies, despite the different degrees of affinity to the sera.

### 2.4. Epitope Analysis of Prudu6 and Glym6

**Figure 5:**
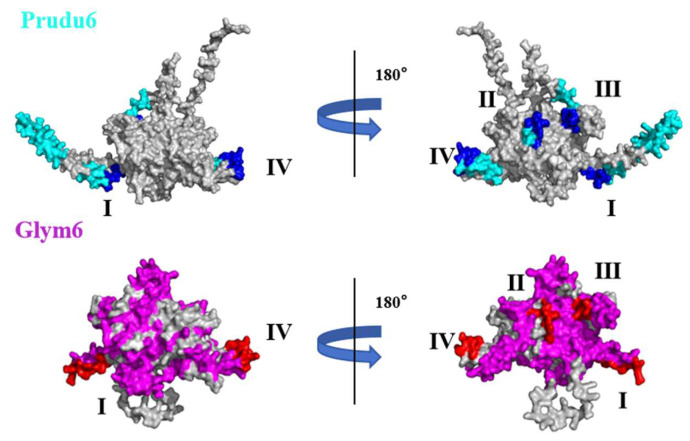
Comparison of the spatial structures of Prudu6 and Glym6 with the identified epitopes colored by cyan and magenta (similar epitopes are represented by blues and reds, respectively). Deeper colors and numbers I–IV correspond to the epitopes in Table 1. The results of the spatial structure comparison are represented by the RMSD value and a smaller RMSD value means a higher similarity [44].

The sensitization of allergens is closely related to their structure, especially the epitope, which is the specific structure identified by IgE in the sera of patients with allergy. Collected from IEDB, the identified epitopes were labeled on the linear structure and conformational structure of the allergens. As shown in Figure 5, the epitope structures were distributed on the surfaces of the allergens that are variable and solvent accessible [43]. The obvious advantage in terms of the quantity of epitope structures for Glym6 indicated its stronger sensitization potential, so that Prudu6 showed a lower inhibition rate in the soybean group (Figure 4B).

**Figure 6:**
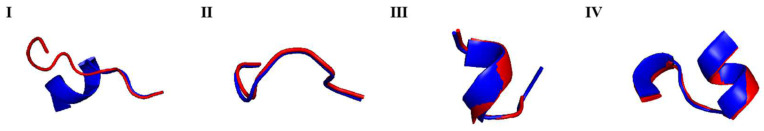
Epitope structures of Prudu6 and Glym6. Prudu6 is colored by blue and Glym6 is colored by red.

In order to identify the core structure causing the cross-reactivity, we screened out four groups of epitopes from the correspondence of Prudu6 and Glym6 in their linear structure and conformational structure with the calculated sequence similarity and RMSD value (Table 1). From Figure 6, we could notice that the first one had a low similarity in the sequence and spatial structure (lower than the whole allergen), so that it was excluded and not analyzed in the following. The remaining three groups of co-epitope structures were different from each other in the structure composition (Figure 6). The second epitope group is composed entirely of the random coil. To our surprise, as a highly variable structure, it is the most similar group in two aspects. The main composition of the third group is the β-fold, while the fourth group is mainly composed of the α-helix. These structures can form hydrogen bonds around their surroundings and make the protein stable, which makes them difficult to affect by external factors and destroy. Such a consistent property enables similar structures to exist stably in proteins. All in all, the high similarity in both structure and sensitization of the three epitopes leads us to infer that they are the core structure of the cross-reactivity between Prudu6 and Glym6.

## 3. Discussion

In the previous literature survey, there were three main methods for Prudu6 purification, including column chromatography, cryoprecipitation and isoelectric precipitation, and the first way was the most commonly used method [22]. Sathe et al. [22] used two-step column chromatography, including the DEAE DE-53 anion exchange column and gel filtration column, to purify Prudu6, which was time-consuming and complex. Through our optimized method, Prudu6 (purity > 85%) was purified with simplicity and at a relatively low cost.

Like almond, soybean can also undergo extensive cross-reactivity with a great diversity of food. Kim et al. [45] discovered the IgE cross-reactivity of soybean with walnut and soybean in children with food allergies. Although the concept of cross-reactivity in relation to almond and soybean has been widely discussed in the literature, this information usually did not involve data about the specific identified allergens or epitopes.

As we expected, the extracts of almond and soybean showed significant binding reactions to both groups of sera, and Prudu6 and Glym6 played important roles in them (Figure 3 and Figure 4). Interestingly, in the competitive inhibition test, Prudu6 had almost no inhibitory effect on Glym6; hence, both showed extremely similar inhibitory curves for the serum of patients allergic to almond. However, when it came to the sera from soybean-allergic patients, Prudu6 was strongly suppressed by Glym6. This difference might imply their similar epitope structure, as well as the conservative epitope structure only found in Glym6 (Figure S3).

In this study, we confirmed the cross-reactivity between Prudu6 and Glym6, and we simultaneously proposed the related core epitopes, which may be the main epitopes identified by IgE from almond-sensitized patients (Figure 5). However, these epitopes seemed less effective in the soybean group, which indicated that the epitopes with more significant sensitization to patients allergic to soybean may exist in the remaining epitope structures and await exploration (Figure 4B).

However, the epitope research on the allergen Prudu6 of almonds is not sufficient, which may lead to certain limitations in the epitope information we have collected. As one of the main allergens in soybeans, the epitope structure of Glym6 has been widely studied, including epitope studies involving cross-reactivity. In previous research, linear epitopes of Glyn6.0201 (GSNILSGFAPEF) and Glym6.0501 (GSVLSGFAPEF) with partial overlap were considered to have a high cross-reaction potential with globulins in peanut [46]. This group of epitopes was also focused on in our research (group III), which indicated that this epitope might be the core epitope that causes soybean to trigger extensive cross-reactivity.

Studying the similar structures among allergens to find regular conclusions is the pursuit of researchers in the field of allergy. The study of these similar epitope structures can provide assistance for the prediction of allergens and related diagnosis and treatment. Studying allergens classified by family with a certain basis of structural similarity is a common method in the field of cross-reactivity research. Bueno-Díaz et al. [37] summarized the structural and immunological characteristics of twelve allergens from the 2S albumin family and confirmed the clinical relevance of their cross-reactivity potential. Although the cross-reactivity and relative epitopes of two 11S globulins, Prudu6 and Glym6, were explored in this study, the limited data was not sufficient to draw convincing regular conclusions. It is anticipated that more experiments involving globulins’ reactivity can be performed to obtain related details.

## 4. Materials and Methods

### 4.1. Human Sera

Sera were obtained from patients with convincing histories of almond allergy or soybean allergy, after informed consent was obtained, or were purchased from PlasmaLab International (Chongqing Manuik Technology Co., Ltd., Chongqing, China). Detailed information can be found in the Supplementary Materials (Tables S1 and S2).

### 4.2. Almond and Soybean Protein Extracts

The ground paste was defatted with acetone at room temperature for 3 h to obtain defatted powder, which was stored at −20 °C for later use. Protein extracts were obtained from defatted flour by extraction with 0.05 mol/L Tris-HCl buffer (pH 8.0) at a ratio of 1:5 (*w*/*v*). After being magnetic stirred at room temperature for 2 h, the extracts were collected after centrifugation at 5000 *g* for 20 min (room temperature).

### 4.3. Purification of Prudu6 and Glym6

The extract was subjected to staged precipitation with ammonium sulfate. The supernatant component was obtained after centrifugation when the saturation of the ammonium sulfate was 70%. After dialysis, anion-exchange chromatography of the supernatant component was performed in a DEAE-Sepharose Fast Flow column, washing with gradient elution at a flow rate of 1.5 mL/min, and the concentration of NaCl increased from 0 M to 0.3 M in the Tris-HCl buffer (50 mM, pH 8.0). Fractions were collected and analyzed by SDS-PAGE to find Prudu6 concentrated in which peak. Glym6 was prepared according to the procedure of Thanh [47].

### 4.4. SDS-PAGE

The diluted protein was mixed with loading buffer and heat-denatured onto the polyacrylamide gel for electrophoretic separation. Following electrophoresis, the gel was stained with Coomassie blue and subsequently decolored. The protein band patterns were visualized and analyzed by densitometry using Quantity One software (version 4.6 Bio-Rad Laboratories, Inc., Hercules, CA, USA).

### 4.5. IgE-Banding Capacity

The IgE-binding capacity of the allergens was assessed by Western blot (WB). After SDS-PAGE, the separated proteins were transferred onto a polyvinylidene fluoride (PVDF) membrane (Bio-Rad Co., Ltd., Shanghai, China), which was then blocked with 5% bovine sera albumin (BSA) for 2 h at room temperature. Subsequently, it was incubated overnight at 4 °C with pooled human sera (1:100 dilution in TBST) from allergic patients. The IgE-binding proteins were detected using horseradish peroxidase (HRP)-conjugated goat anti-human IgE antibodies (Bio-Rad Laboratories, Inc., Hercules, CA, USA), followed by streptavidin (Neobioscience, Beijing, China) enhancement. (After each step was completed, five rounds of cleaning were carried out using TBST and five minutes per time to stop the reaction.) The immunoreactive bands were visualized using an enhanced chemiluminescence (ECL) reagent (Sangon Biotech Co., Ltd., Shanghai, China). After reacting for a minute, the chemiluminescent signals were captured and quantified using an ImageQuant LAS 500 imaging system (Beijing IMH-bio Technology Limited, Beijing, China).

### 4.6. Identification of Purified Protein

Bands from the SDS-PAGE exceeding 60 kDa were excised for identification. After reduction, alkylation, in-gel digestion, and desalting [48], one-dimensional chromatographic column analyses were performed by applying an EASY–nLC 1200 system coupled to a Q Exactive mass spectrometer (Thermo Scientific, CA, USA). The resulting spectra were searched against the UniProt database by Proteome Discoverer software (version 2.5, Thermo Scientific, CA, USA).

### 4.7. ELISA Inhibition Assays

An indirect competitive enzyme-linked immunosorbent assay (ELISA) was performed for the IgE-binding inhibition analysis. Sera from allergic patients (1:100 dilution in PBS) were preincubated with Prudu6 or Glym6 at different concentrations for 1 h at 37 °C. Then, the mixture was transferred to a 96-well plate incubated with Prudu6 or Glym6 at 4 °C overnight and incubated at 37 °C for 1 h. Next, 100 μL of goat anti-human IgE-HRP conjugate was added and incubated for 1 h. Then, 100 μL of HRP-labeled avidin was added and incubated at 37 °C for 1 h. After the color development was terminated by adding 50 μL of 2 M concentrated sulfuric acid (Yeasen Biotechnology Co., Ltd., Shanghai, China) to each well (about 5 min), the absorbance of the samples was determined at 450 nm using a microplate reader (model 1860, Bio-Rad Co., Ltd., Shanghai, China). Washing was performed five times (5 min per time) with PBS at the end of each step. Allergen coated with PBS was regarded as the negative control and allergen coated with sera was regarded as the positive control. The percentage of inhibition of antibody binding was calculated as follows: Inhibition (%) = (1 − (A1 − A0)/AC − A0)) × 100, where AC—OD450 of the positive control; A1—average OD450 of the test samples; A0—OD450 of the negative control.

### 4.8. Structure Characterization and Epitope Analysis

An MOS-450 spectropolarimeter (French Bio-Logic SAS, Claix, France) was used for the analysis of the secondary structures by circular dichroism (CD) spectroscopy. The concentration of the allergens was 0.2 mg/mL (dissolved in PBS) for the far-ultraviolet region ranging from 190 to 240 nm. The ultraviolet (UV) absorption was evaluated using a UV spectrophotometer (Purkinje General, Beijing, China). The samples were homogeneously mixed at a concentration of 0.2 mg/mL in PBS and then scanned from 250 to 300 nm. The amino acid sequences [UniProt ID E3SH28 and P04776] were obtained from UniProt and the amino acid sequence of Prudu6 was inputted to the SWISS-MODEL to select the highest-scoring protein as the tertiary structure sample (Figure S2). The Glym6 structure was derived from an experimental crystal structure [PDB entry 1FXZ]. Identified linear epitopes of Prudu6 and Glym6 were gained from the IEDB website (Tables S4 and S5). The similarity in terms of the sequence was compared by Clustal W and ESPript (3.x), while the tertiary structure was analyzed using PyMOL (3.1.0). Similar epitopes were screened based on the correspondence relationship in the comparison of the linear structure and conformational structure.

## Figures and Tables

**Table 1:** Corresponding epitopes in the sequence and spatial structure.

	Prudu6	Glym6	Sequence Similarity	RMSD
I	^113^EESQQSSQQG^122^	^112^EEPQQPQQRG^121^	40%	3.740
II	^232^HNQLDQNP^239^	^172^ENQLDQMP^179^	75%	0.087
III	^288^GNNVFSGF^295^	^217^GGSILSGF^224^	62.5%	0.218
IV	^516^VLANAYQISREQ^527^	^459^VIQHTFNLKSQQ^470^	58.33%	0.199

## Data Availability

All data generated and analyzed during this study are included in this published article and its Supplementary Materials.

## Supplementary Materials

The following supporting information can be downloaded at https://www.mdpi.com/article/10.3390/ijms26115425/s1.

## Abbreviations

The following abbreviations are used in this manuscript:

WB	Western blot
ELISA	enzyme-linked immunosorbent assay
PBS	phosphate-buffered saline
CD	circular dichroism
PVDF	polyvinylidene fluoride
BSA	bovine sera albumin
UV	ultraviolet
M	mol/L
