# Supplementary material for: Purification of Prudu6 from Almond and Its Cross-Reactivity with Glym6 from Soybean
Source: Int J Mol Sci. 2025 Jun 5;26(11):5425. doi: 10.3390/ijms26115425 (PMC12156343; doi:10.3390/ijms26115425)
Supplement: Supplementary file 1 [file ijms-26-05425-s001.zip › ijms-3662627-supplementary.pdf]

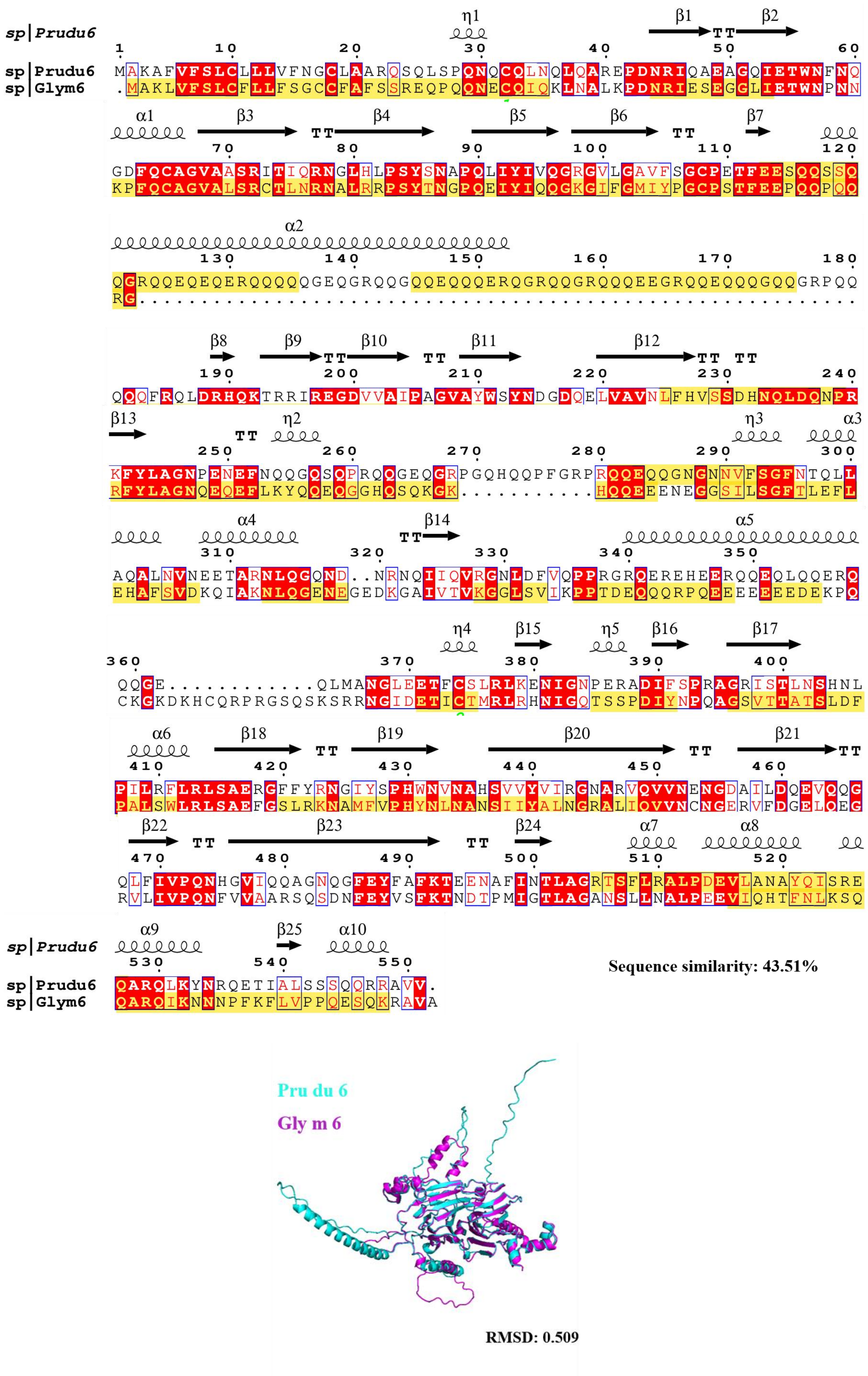

Figure S1. Comparison of spatial structure and sequence with identified epitopes colored by yellow.

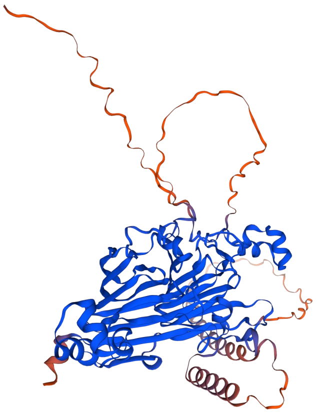

Figure S2. The confidence levels of the predicted Pru du 6 structure. Blue mean the confidence of the region is high (>90) and red one showed lower confidence (<50).

I II III IV

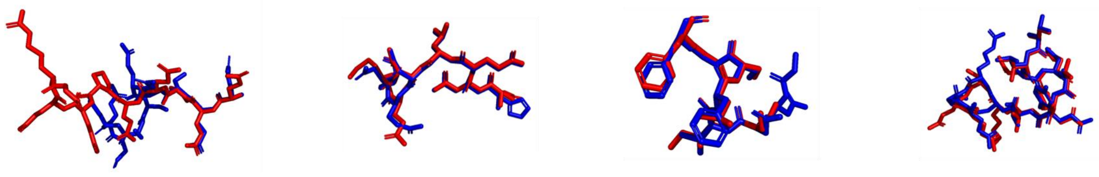

Figure S3. Epitope structure of Prudu6 and Glym6 in stick

model Table S1. Information of patients allergic to almond

Number	Gender	Age	Clinical symptoms	Total IgE level （KU/L）	Specific IgE level（KU/L）
1	Female	21	Allergic rhinitis, no immunotherapy	2516	41.2

Table S2. Information of patients allergic to soybean

Number	Gender	Age	Clinical symptoms	Total IgE level （KU/L）	Specific IgE level（KU/L）
1	Female	27	Allergic rhinitis, yes immunotherapy	7707.573	72.333
2	Male	27	Allergic rhinitis, no immunotherapy	579	13.3
3	Male	29	Allergic rhinitis, yes	696.63	7.63

			immunotherapy		
4	Male	25	Allergic rhinitis, no immunotherapy	ND*	8.94
5	Male	24	Allergic rhinitis, yes immunotherapy, food IgG positive	1345	22.2
6	Male	54	Allergic rhinitis, yes immunotherapy, Multiallergic	2730.248	6.336
7	Female	61	Allergic rhinitis	1501.217	8.254
8	Female	25	Allergic rhinitis, no immunotherapy	1223.383	7.701
9	Male	21	Allergic rhinitis, no immunotherapy	ND*	8.09
10	Male	28	Allergic rhinitis	21940	8.27

ND* indicates unknown。

Table S3. Peptide information was identified by MS

Accession	Coverage	Peptides	Unique Peptides	Score Sequest
E3SH28	75	30	30	4051.61
A0A5E4GEN6	32	11	11	46.73
A0A5E4EE27	27	11	11	46.18
A0A5E4EZP4	14	8	8	23.14
A0A5E4G4E7	39	1	1	21.23
A0A5E4F7J3	8	4	4	13.25
A0A5E4EYX0	9	2	2	11.07
A0A5E4F5R8	12	4	4	10.32
A0A5E4FUS1	7	3	3	9.8
A0A5E4G4C3	2	2	2	8.33

Table S4. Identified linear epitopes of Pru du 6 from IEDB

Epitope	Start	End
EESQQSSQQGRQQEQ	113	127
QQQQQQQFRPSRQEG	113	127
QGRQQEQEQERQQQQ	121	135

QQEQQQERQGRQQGR	145	159
QGRQQGRQQQEEGRQ	153	167
QQEEGRQQEQQQGQQ	161	175
LFHVSSDHNQLDQNP	225	239
GNNIFSGFDTQLLAQ	225	239
QQEQQGSGNNVFSGF	281	295
RTSFLRALPDEVLAN	505	519
PDEVLANAYQISREQ	513	527

Table S5. Identified linear epitopes of Gly m 6 from IEDB

Epitope	Start	End
MAKLVLSLCFLLFSG	1	15
KLVLSLCFLLFSGCF	3	17
CFLLFSGCFALREQA	9	23
LFSGCFA	12	18
SGCCFAF	14	20
CFAFSF	17	22
LREQAQQNECQIQ	19	31
FSFREQPQ	20	27
QPQQNECQIQ	25	34
NRIESEG	40	46
PDNRIE	41	46
RIESEG	44	49
SEGGFI	44	49
KPFQCAGVALSRCTL	57	71
KPFQCAGVA	57	65
ALSRCTLNRNALRRP	65	79
RNALRRPSYTNGPQE	73	87
YTNGPQEIYIQQGNG	81	95

RPSYT	81	85
NGPQEIYIQQGNGIF	83	97
NGPQE	86	90
YIQQGNGIFGMIFPG	89	103
EIYIQQG	90	96
QQGNGIFGMIFPGCP	91	105
YIQQGSGI	92	99
FGMIFPGCPSTYQEP	97	111
IFPGCPSTY	100	108
IYPGCPSTFEE	103	113
EPQESQQ	110	116
QESQQRG	112	118
EPQQKG	113	118
EPQQKGQSSRPQDRH	113	127
EPQQPQQRG	113	121
QQKGQSSR	115	122
GRSQRPQDRHQ	118	128
QRPQDRHQK	121	129
GQSSRPQD	121	128
DRHQKIYHF	125	133
PQDRHQ	126	131
QKVHRF	128	133
VHRFREGDLIAV	130	141
IYHFRE	130	135
GDLIAVPTGVAWWMYNNE	136	153
IAVPTGF	139	145
IAVPTG	142	147
PTGVAW	142	147
FQNQLDQ	168	174

ENQLDQ	169	174
NQLDQMPRRFYLAGN	170	184
SLENQLDQMPRRFYLAGNQEQEFLKYQQEQG	170	200
PRRFY	176	180
DQMPRRFYLAGNQE	176	189
RRFYLAGNQEQEFL	177	190
RFYLAGNQEQEFLKY	178	192
GNQEQ	183	187
EQEFLKYQQQQQGGS	186	200
EQEFL	186	190
EFLKYQQQQQ	188	197
FLKYQQQ	189	195
FLQYQPQ	189	195
LKYQQQQQGGSQSQK	190	204
EFLKYQQEQG	191	200
FLKYQQE	192	198
QQQQGGSQSQKGKQQ	194	208
QQQGGSQ	195	201
QQEQGGHQ	196	203
GSQSQKG	199	205
HQSQKG	202	207
KHQQEE	208	213
GSNILSGFAPEF	214	225
GGSILSGFTLEFL	217	229
SGFAPEFLKEAFGVN	219	233
SGFTLEFLEHAFSVD	222	236
GFTLEFLEHAFSV	223	235
GFTLEF	223	228

FLEHA	228	232
EHAFSVD	230	236
NLQGE	242	246
QGENE	244	248
SGAIVTVKGGLRVTA	249	263
GAIVTVKGGLSVI	253	265
KGGLRV	256	261
GGLRVTAPAMRKPQQ	257	271
GGLSVISP	259	266
LSVIKP	262	267
PPTEEQQ	266	272
PTDEQQQ	268	274
QEEDDDDEEEQPQCVE	271	286
QRPQEE	274	279
RPEEEEKP	274	281
EEKPDCDE	278	285
EEEEDEK	281	287
QCVETDKGC	283	291
EKPQC	286	290
QSQSRN	291	296
KDKHC	293	297
ETICT	314	318
TSSPDIF	314	320
GSITTATSLDFPALW	329	343
LDFPALWLLKLSAQY	337	351
LKLSAQYGSLRKNAM	345	359
SLRKNAMFVPHYTLN	353	367
MFVPHYTLNANSIIY	359	373
VPHYTLNANSIIYAL	361	375

NANSIIYALNGRALV	367	381
NSIIYALNGRALVQV	369	383
FVPHYNL	370	376
VIQHTFNLKSQQARQ	449	463
QHTFNLKSQQARQVK	451	465
KSQQARQVKNNNPFS	457	471
FLVPPQESQK	482	491
